# The biochemistry of melanogenesis: an insight into the function and mechanism of melanogenesis-related proteins

**DOI:** 10.3389/fmolb.2024.1440187

**Published:** 2024-08-20

**Authors:** Feifei Wang, Wenjing Ma, Dongjie Fan, Jing Hu, Xiaohong An, Zuding Wang

**Affiliations:** ^1^ Yunnan Characteristic Plant Extraction Laboratory, Yunnan Yunke Characteristic Plant Extraction Laboratory Co., Ltd., Kunming, China; ^2^ Yunnan Botanee Bio-Technology Group Co., Ltd., Kunming, China; ^3^ State Key Laboratory of Natural Medicines, School of Traditional Chinese Pharmacy, China Pharmaceutical University, Nanjing, China; ^4^ Shanghai Jiyan Bio-Pharmaceutical Co., Ltd., Shanghai, China

**Keywords:** melanocytes, melanin, melanosomes, melanogenesis, function, mechanism

## Abstract

Melanin is an amino acid derivative produced by melanocyte through a series of enzymatic reactions using tyrosinase as substrate. Human skin and hair color is also closely related to melanin, so understanding the mechanisms and proteins that produce melanin is very important. There are many proteins involved in the process of melanin expression, For example, proteins involved in melanin formation such as p53, HNF-1α (Hepatocyte nuclear factor 1α), SOX10 (Sry-related HMg-Box gene 10) and pax3 (paired box gene 3), MC1R(Melanocortin 1 Receptor), MITF (Microphthalmia-associated transcription factor), TYR (tyrosinase), TYRP1 (tyrosinase-related protein-1), TYRP2 (tyrosinase-related protein-2), and can be regulated by changing their content to control the production rate of melanin. Others, such as OA1 (ocular albinism type 1), Par-2 (protease-activated receptor 2) and Mlph (Melanophilin), have been found to control the transfer rate of melanosomes from melanocytes to keratinocytes, and regulate the amount of human epidermal melanin to control the depth of human skin color. In addition to the above proteins, there are other protein families also involved in the process of melanin expression, such as BLOC, Rab and Rho. This article reviews the origin of melanocytes, the related proteins affecting melanin and the basic causes of related gene mutations. In addition, we also summarized the active ingredients of 5 popular whitening cosmetics and their mechanisms of action.

## 1 Introduction

The type, quantity, and distribution of melanin are the primary factors influencing human skin color. Melanin is highly concentrated in the skin, hair, mucous membranes, and other tissues (Lapierre-Landry et al., 2018). Melanin is generated by melanocytes (MC), which arise from neural crest cells (NCCs) and are mostly located in the basal layer of the mammalian epidermis and hair follicles.

In the typical human epidermis, 40 keratinocytes (KCs) cluster around 1 MC, and MC will unite keratinocyte (KC). KCs connective dendritic branches act as conduits for mature melanosome trafficking to the KC (Moreiras et al., 2021). The research that is currently accessible describes transplantation of epidermal cultivated melanocytes or melanocyte-keratinocyte suspension as a unique therapy option for vitiligo (Nilforoushzadeh et al., 2022).

Melanin is produced in melanosomes, and three enzymes, TYR, TYRP-1, and TYRP-2, play important roles in melanin formation (Lai et al., 2018). The substrate tyrosine gets converted to dopaquinone by the rate-limiting enzyme TYR, which can transform to colorless dopachrome. Some proteins, including as MITF, PKC, and HNF-1α, regulate melanin production by altering TYR. However, the majority of proteins, including PAX3 and SOX10, p53, and the melanocortin-1 receptor MC1R, regulate melanin production by directly or indirectly affecting MITF. Others include OA1, melanophilin Mlph, Protease activated receptor-2, PAR-2, and other similar proteins, which are primarily involved in the process of melanin expression via influencing melanosome trafficking.

In addition to changing human skin color and hair color, melanin also has some very valuable advantages. Melanin not only absorbs UV and visible light but also has free radical scavenging and antioxidant capacity, it protects cells from toxic damage and boundaries the damaging effects of UV on cellular macromolecules. This prevents DNA damage and pathogenic mutations from UV radiation on the skin (Swope and Abdel-Malek, 2018).

## 2 The source of melanin and melanocytes

### 2.1 Different types of melanocytes

All pigment cells in vertebrates, with the exception of retinal pigment epithelial cells, are produced from neural crest cells (NCCs) (Alkobtawi et al., 2018). Melanocytes in the brain come from cranial neural ridge cells, whilst those in the limbs and trunk originate from trunk neural crest cells (Bronner and LeDouarin, 2012; Vega-Lopez et al., 2018). NCCs originate from the dorsal border of the neural tube, which is created by ectoderm, are exclusive to vertebrates. Numerous derivatives, including the pigmentation of the skin, adrenomedullary cells, facial bone and cartilage, and the sensory and autonomic ganglia of the peripheral nervous system, are formed by them. A subgroup of embryonic stem cells known as neural crest cells (NCCS) can come from a variety of cell types (Noisa and Raivio, 2014).

Early in the development of the vertebrate embryo, the elevated nerve folds of the ectodermal nerves close to form neural tubes. Numerous neural crest cells can be found in the dorsal area of the neural tube. The neural ridge cells enter a migration staging area (MSA) at the beginning of the complicated differentiation process, where they choose to migrate dorsolaterally (between the outer embryo and the body segment) or ventrally (between the neural tube and the body segment). According to their final differentiation sites, neural ridge cells can currently be classified as cranial ridge, vagus ridge, trunk ridge, and caudal sacral ridge.

There are two forms of pigment cells in the embryonic stage, one is formed by the migration of neural crest cells with pigment cell properties along the dorsolateral side, and the other is formed by the neural crest cell subcellular Schwann cell precursor migrating along the ventral side after receiving some signal induction. Studies suggest that the differentiation of pluripotent neural crest cells is specialized before they migrate out of the neural tube, rather than being influenced by the migration environment. Early migrating neural crest cells localize to the sympathetic ganglia, intermediate migrating cells localize in the dorsal root ganglia, and the last migrating cells form pigment blasts under the dermal sarcomere and ectoderm. These pigment precursor cells then differentiate through specific developmental pathways to form pigment cells (Jacobs-Cohen et al., 2002; Dupin and Le Douarin, 2003; Thomas and Erickson, 2008; Sommer, 2011).

Melanocytes are mainly divided into two categories: skin melanocytes and non-skin melanocytes. The source of melanocytes in the skin is mainly the neural crest cell subcellular Schwann cell precursors (SCPs), formed after being induced by some kind of signal.

### 2.2 Different transport modes of melanosomes

According to the current research progress, the synthesis process of melanin in the organism has been basically determined, and how melanosomes are transported to keratinocytes, four possible ways have been proposed, but no one is conclusive (Bento-Lopes et al., 2023). The first hypothesis is that surrounding keratinocytes would exert direct cellular phagocytosis on melanocyte dendrites containing melanosomes (Wolff, 1973; Okazaki et al., 1976; Yamamoto and Bhawan, 1994). The second hypothesis is that melanocytes shed melanosomes, followed by cellular phagocytosis of the shed melanosomes by keratinocytes (Cerdan et al., 1992; Ando et al., 2011; Ando et al., 2012). Form the third hypothesis is horniness cells and melanocytes occur between the cell membrane fusion, so that the formation of melanin body directly transferred to the cutin cell (Scott et al., 2002; Tarafder et al., 2014; Singh et al., 2017; Hurbain et al., 2018). Fourth, melanosome enters the intercellular space from melanocytes by exocytosis and enters keratinocytes by endocytosis (Swift, 1964; Tarafder et al., 2014; Correia et al., 2018; Hurbain et al., 2018). These pathways are not mutually exclusive, they may coexist, and melanosome transport may be completed through multiple pathways.

### 2.3 Different types of melanin

Melanin derived from tyrosine and other similar phenolic chemicals form the widely dispersed phenolic biopolymer. According to the different synthetic pathways and intermediate metabolites, melanin can be mainly divided into eumelanin, pheomelanin and allomelanin, and its sources can be bacteria, fungi, plants or animals (Wakamatsu and Ito, 2023).

L-tyrosine is converted by tyrosinase to L-3,4-dihydroxyphenylalanine (L-DOPA) and L-DOPA to dopaquinone. Dopaquinone then proceeds through a sequence of reduction and oxidation processes to yield 5,6-dihydroxyindole and 5,6-dihydroxyindole-2-carboxylic acid, which are the building blocks of eumelanin.

**FIGURE 1:**
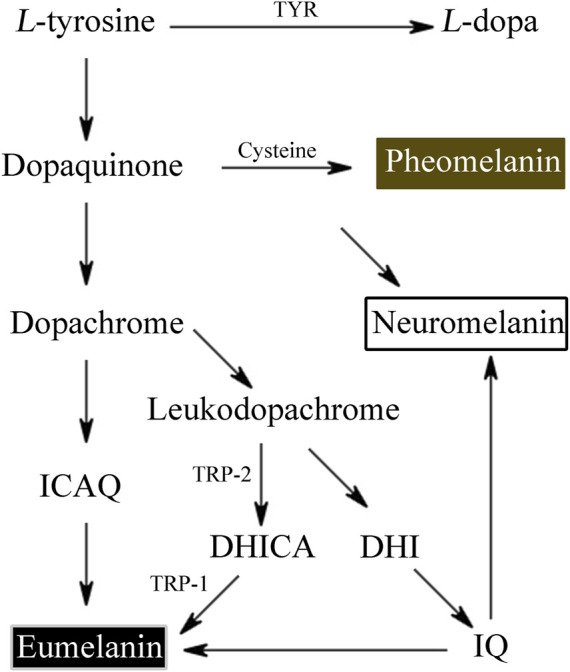
The process of tyrosine derivative of melanin.

Tyrosinase converts the amino acid L-tyrosine into the eumelanin that makes up human skin. Phaeomelanin is a cysteine derivative that is primarily responsible for the color of red hair, as well as other pigmentation. Neuromelanin is found in the brain, studies have been conducted to investigate its efficacy in treating neurodegenerative diseases such as Parkinson’s disease (Nagatsu et al., 2022) (Figure 1). Neuromelanin can participate in neuroprotective or toxic processes that occur in Parkinson’s disease. Neuromelanin can prevent the toxic accumulation of active compounds derived from catechins, and can also provide neuroprotective effects by combining reactive/toxic metals to produce stable and non-toxic complexes (Zucca et al., 2023). The eumelanins that we’re most concerned about are the two most common types—brown eumelanin and black eumelanin, because human skin exhibits different colors based on differences in the amount of brown and black pigment and the ratio of the two. Brown eumelanin mainly controls the color of brown, showing differences in shades from yellow to brown as the content of brown eumelanin varies. Depending on the amount of melanin in the body, it will appear light gray, dark gray, or black.

## 3 Melanin-related proteins

**FIGURE 2:**
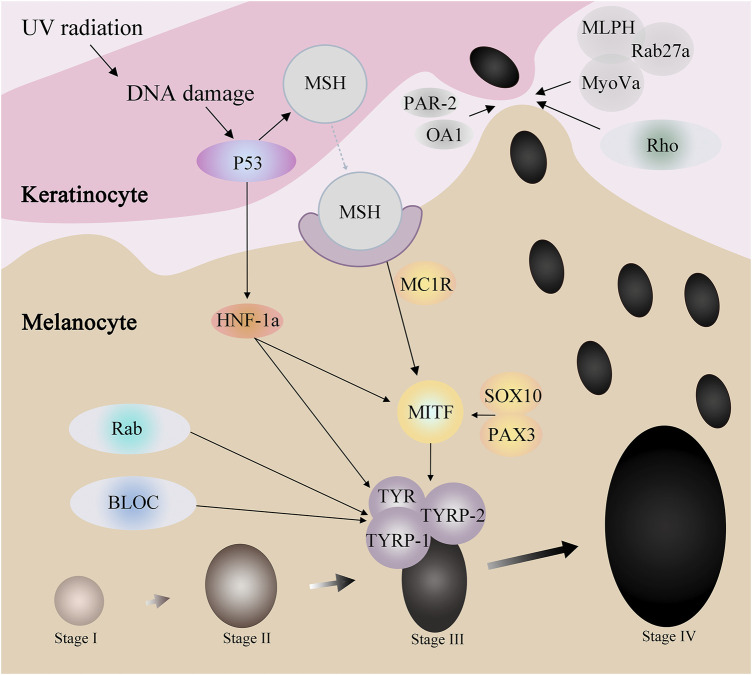
Proteins related to melanogenesis and melanosome transport.

Melanocytes produce melanosomes, which are then transported to adjacent keratinocytes, ascending in layers, and eventually visible in the human epidermis. In this process, many ions interact with proteins to affect the process, some of which affect the production of melanosome, and some of which regulate the transport of melanosome. And some protein family not only affects the generation of melanin small body, also in the transport of melanin body play a role (Bento-Lopes et al., 2023) (Figure 2).

### 3.1 Proteins involved in melanogenesis

Proteins such as p53, HNF-1α, SOX10, PAX3, and MC1R affect MITF through a series of regulations, thereby affecting tyrosinase activity to regulate melanin production. For example, when the skin is stimulated by UV radiation, the DNA damage caused can activate p53. On the one hand, p53 can promote the expression of HNF-1α, while HNF-1α can promote the expression of MITF, and on the other hand, it can directly act on MITF. MITF, by adjusting the TYR and TRP - 1, the expression of TRP - 2 to participate in the generation of melanin.

#### 3.1.1 P53

The human p53 gene is located on chromosome17p13.1, with a total length of 1620 kb. It contains 11 exons and 10 introns, encoding a 393 amino acid nuclear phosphorylated protein with a molecular weight of 53kD. P53 protein has a clear binding site with the regulatory sequence of TRP1 gene, which directly regulates the synthesis of melanin. Uv irradiation can stimulate the upregulation of P53 protein in keratinocytes, thereby promoting the expression of keratinocyte cytokines such as proopiomelanocortin POMC (Cui et al., 2007). POMC can bind to receptors on the surface of melanocytes to promote the proliferation of melanocytes and the synthesis of melanin. Expression of endothelin ET and stem cell factor SCF, which are associated with melanin synthesis, was higher in skin pigmented keratinocytes than in other tissues (Imokawa et al., 1997; Hachiya et al., 2004). Treatment of cultured tissues and mice with p53 siRNA downregulated ET and SCF expression (Murase et al., 2009).

As a powerful tumor suppressor, p53 inhibits tumor growth in several ways. As a transcription factor, p53 coordinates the expression of target genes and can promote cell cycle arrest, apoptosis, DNA repair, etc., (Kastan et al., 1995; Fridman and Lowe, 2003; Williams and Schumacher, 2016; Kastenhuber and Lowe, 2017). However, current studies have shown that P53 is related to the deterioration of melanoma, and the higher the malignant degree, the higher the positive rate of p53 expression, which contradicts p53 as a tumor suppressor, but the specific mechanism is still unclear.

#### 3.1.2 HNF-1α

Hepatocyte nuclear factor 1α (HNF1-α) gene is located on human chromosome 12q24.31, a POU-homeodomain family transcription factor, expressed predominantly in the liver, and it regulates many aspects of hepatocyte function (Shih et al., 2001; Costa et al., 2003; Odom et al., 2004). In melanocytes, HNF1A is activated by p53, which not only binds to the enhancer of MITF and enhances MITF transcription (Schallreuter et al., 2008), also can bind to the promoter of the tyrosinase gene to activate the transcription of tyrosinase, thus further promoting the transcription of tyrosinase gene (Schallreuter et al., 2003).

Mutations in the HNF-1α gene cause Maturity-onset diabetes of the young (MODY) (Yamagata et al., 2007). Large-scale genetic studies have clarified that the common variants of HNF1α and HNF4α genes are also associated with type 2 diabetes, suggesting that they are involved in the pathogenesis of both diseases (DeForest et al., 2023). Twenty-seven single nucleotide polymorphisms (SNPS) in the HNF-1α gene are associated with an increased risk of coronary artery disease.

#### 3.1.3 SOX10 and PAX3

SRY-box 10 (Sox10) located in human chromosome 22q13.1 It is a member of the SRY-related HMG-box (SOX) family of transcription factors and a regulatory molecule that plays an important role in the development of the neural crest and the peripheral nervous system (Britsch et al., 2001; Hong and Saint-Jeannet, 2005; Bondurand and Sham, 2013). PAX3 located in human chromosome 2q36.1 It contains 10 exons and is a member of the paired box transcribed silver PAX family. SOX10 and PAX3 are widely expressed in NCC and NC-derived tissues, and play important roles in the early development of melanocytes (D. S. Wilson et al., 1995; Takebayashi et al., 1996; Lang et al., 2000; Hollenbach et al., 2002; Yasumoto et al., 2002). Without the regulation of SOX10 and PAX3, it is difficult for MITF to affect the transcription of TYR and thus the differentiation of melanocytes. Moreover, SOX10 can also directly induce the expression of DCT121 and TYR78. PAX3 can upregulate the transcriptional activity of TYRP1, thereby affecting the production of melanin (Poulat et al., 1995)^.^ A large number of experiments have confirmed (Bertolotto et al., 1998; Watanabe et al., 1998; Bondurand et al., 2000; Potterf et al., 2000; Yasumoto et al., 2002; Ludwig et al., 2004). In melanocyte development, SOX10 increases MITF transcription 100-fold after binding to a highly conserved sequence in the MITF promoter, a mechanism that is enhanced by PAX3, but the ability of PAX3 alone to activate MITF transport is weak (Hershey and Fisher, 2005; Sánchez-Mejías et al., 2010). The transcriptional activation of TYRP2/DCT by MITF requires a synergistic effect with SOX10 (Bertolotto et al., 1998; Yasumoto et al., 2002; Ludwig et al., 2004), indicating that SOX not only induces MITF expression but also regulates the transcription of other genes specifically expressed in melanocytes during melanocyte development.

Mutations in PAX3 cause Waardenburg syndrome type 1 and Waardenburg syndrome type 3 (Cao and Wang, 2000; Pingault et al., 2002; Mollaaghababa and Pavan, 2003; Baxter et al., 2004), At the same time, PAX3 plays an important role in the regulation of normal cell apoptosis, proliferation and differentiation. Once PAX3 is lacking, the balance between cell proliferation and differentiation will be broken (Cao and Wang, 2000). SOX10 mutations cause WS2E (Gether, 2000; Mollaaghababa and Pavan, 2003; Baxter et al., 2004).

#### 3.1.4 MC1R

The melanocortin (MC) receptor family is the smallest member of the class A (rhodopsin-like) family of G-protein coupled receptors (GPCRs) (Gether, 2000; Montero-Melendez, 2015). And consists of five members: MC1R, MC2R, MC3R, MC4R, and MC5R with varying tissue expression and functions.

The human MC1R is 317 amino acids (García-Borrón et al., 2005), it was originally identified and cloned by two independent groups (Chhajlani and Wikberg, 1992; Mountjoy et al., 1992)^,^ and mapped to chromosome 16q24.3 (Gantz et al., 1994). The receptor is primarily located on melanocytes and transformed melanoma cells (Ghanem et al., 1988; Siegrist et al., 1989; Donatien et al., 1992; Siegrist et al., 1994). MC1R protein expression is typically low, with an estimated 700 protein units expressed per melanocyte and somewhat higher numbers on melanoma cells (Donatien et al., 1992; Roberts et al., 2006). In humans, MC1R is activated by KC-derived α-MSH, which stimulates MITF and accelerates melanin biosynthesis (Goding and Arnheiter, 2019). It converts the yellow-red sulfur-containing pheomelanin base substance into black-brown eumelanin, leading to deeper pigmentation and promoting the transfer of pigment to KCs (Guida et al., 2022).

Recent scientific evidence suggests that MC1R activation enhances the process of DNA repair, which could potentially prevent melanoma. This is contrary to the previous belief of most researchers that the overexpression of MC1R in early melanoma cells will promote its proliferation. Instead of proliferation, MC1R will play a preventive and repair role (Chen et al., 2017; Castejón-Griñán et al., 2018; Montero-Melendez et al., 2022). Beyond its role in pigmentation, monocytes and macrophages are known to express the MC1R, which mediates anti-inflammatory effects and helps prevent macrophage foam cell production by increasing cholesterol efflux via ATP-binding cassette transporter A1 (ABCA1) and adenosine triphophate (ATP)-binding cassette (ABC) transporter G1 (ABCG1) transporters (Catania et al., 2004; Rinne et al., 2017).

#### 3.1.5 MITF

The Microphthalmia-associated transcription factor (MITF) gene is a basic helix-loop-helix leucine zipper transcription factor (T. Chang et al., 1993) which acts as a reaction substrate of ubiquitin ligase VCHL1 and regulates the expression of key enzymes in melanin synthesis, such as TYR (Shi et al., 2022). It can regulate the expression of TYR, TRP-1 and TRP-2 by binding to the M-box motif in the promoter region (a highly conserved sequence shared by TYR, TRP-1 and TRP-2 in the promoter region, namely 5′-AGTCATGTGCT3′), thereby regulating the production of melanin (Y. S. Hwang et al., 2017). MITF is located on human chromosome 3p13 (Hou and Pavan, 2008). The retinal pigment epithelial (RPE) represents the first site of MITF expression, followed by the expression in the neural crest. The mutation of MITF gene can lead to a series of phenotypic changes in many species, especially in pigment cells. Some mutations can affect RPE, leading to hypopigmentemia and microphthalmia (Nakayama et al., 1998). Loss of MITF gene expression in humans can cause Waardenburg syndrome type I, which is characterized by congenital cataract and nerve deafness. In addition, a few MITF alleles have important regulatory effects on the growth and development of melanocytes, melanin production and transport by affecting osteoclasts and leading to bone sclerosis (Cheli et al., 2010). MITF can regulate a variety of melanosome production related proteins and melanosome transport related proteins, such as TYR, TYRP1, TYRP2, MC1R, KIT, Rab27a, OA1 and so on (Hoek et al., 2008).

MITF haploinsufficiency can lead to Waardenburg syndrome type 2 (Hodgkinson et al., 1998) Mutations in the MITF gene cause Waardenburg syndrome (S. Kumar and Rao, 2012) and Tietz syndrome (S. D. Smith et al., 2000), Both disorders are accompanied by symptoms such as inadequate melanocyte development, insufficient pigmentation of the skin and hair, and deafness. In addition, Tietz syndrome is accompanied by leukemia.

#### 3.1.6 TYR-TYRP1-TYRP2

There are three types of tyrosinase and its related proteins, including: Tyrosinase (TYR), Tyrosinase-related protein-1 (TYRP1), also known as gp75 (Glycoprotein 75), and tyrosinase-related protein-2 (TYRP2), also known as dopachrome tautomerase (DCT) (Villareal et al., 2010). TYR gene, located in human chromosome 11q14.3 (Barton et al., 1988), is a rate-limiting enzyme that controls melanin production and catalyzes the early rate-limiting reaction of melanin production (Kumar et al., 2011). TYRP1 gene, located in human chromosome 9p23 (Box et al., 1998). It is a specific gene product of melanocytes involved in the production of melanin. In addition, TYRP1 has been suggested to function as a 5,6-dihydroxyindole-2-carboxylic acid (DHICA) oxidase in murine melanocytes (Jiménez et al., 1988), and it is also involved in the formation of melanosome structures (Sarangarajan and Boissy, 2001). TYRP2 chromosomal localization to 13q31-q32 (Bouchard et al., 1994). It mediates the tautomerization of the red melanin precursor, L-DOPA chrome to the colorless DHICA. In the absence of DCT, L-DOPA chrome is spontaneously converted to the toxic melanin precursor, DHI (Leonard et al., 1988; Tsukamoto et al., 1992; Kroumpouzos et al., 1994).

In the second stage of melanosome formation, TYR, TYRP1 and TYRP2 are transported into melanosomes (Hellström et al., 2011), Beginning with the melanosome stage III, these enzymes catalyze the conversion of tyrosine to pigment (Kobayashi et al., 1994). TYR gene mutation inhibits the production of TYR, directly affects the production of melanin, and then causes oculocutaneous albinism type 1 (OCA1) (Witkop and Wolff, 1979). TYRP1 gene mutation causes oculocutaneous albinism type 3 (OCA3) (Wakamatsu and Ito, 2023). The TYRP2 gene regulation mechanism is negatively correlated with the melanocyte growth regulation pathway.

### 3.2 Proteins involved in melanin transport

Although the generation of melanosome is the most important step for melanin to reach the skin surface, the transport of melanosome is also an indispensable focus. For example, OA1 and PAR-2 affect melanosome transport by promoting the transport of melanosome from melanocytes to keratinocytes. MLPH does the same, but as a three-protein complex, MLPH-MyoVa-Rab27a, to facilitate melanosomes trafficking.

#### 3.2.1 OA1

The Ocular albinism type 1 (OA1) gene is located on the human chromosome xp22.3-xp22.2 and encodes a 404 amino acid (Shen et al., 2001). OA1 protein is expressed more in the retinal pigment epithelium and skin and less in the brain and adrenal gland (Oetting and King, 1999). OA1 receptor is a typical G protein-coupled receptor, a conserved intact membrane protein with seven transmembrane domains. OA1 protein mainly exists in the endocytic lysosomes of pre-melanosome and the membrane of mature melanosome, connecting intracellular lysosomes and melanosome (Staleva and Orlow, 2006). It may be involved in vesicle trafficking or melanosome sorting (Young et al., 2013). Levodopa (L-DOPA) is a specific ligand for the OA1 receptor (Gross, 2008), it is also a by-product of the melanin biosynthesis pathway. Activation of OA1 receptors by L-DOPA results in secretion of a neurogenic factor by retinal pigment epithelial cells that contributes to normal retinal development (Lopez et al., 2008).

Loss of function due to mutations in the OA1 gene, causing ocular albinism type 1, also known as “Nettleship-Falls syndrome (Oetting, 2002).” It is an inherited X-linked recessive disorder with a higher incidence in males than in females. It causes retinal hypopigmentation, nystagmus, strabismus, foveal hypoplasia, abnormal fiber crossing, and decreased vision (Oetting, 2002), strabismus and fundus pigmentation decreased while skin and hair color remained normal (Lam et al., 1997; Preising et al., 2001; Sallmann et al., 2006).

#### 3.2.2 PAR-2

Protease activated receptor 2 (PAR-2), also known as coagulation factor II (thrombin receptor-like 1, F2RL1), which is encoded by F2RL1 gene in human chromosome 5q13.3, can regulate the body’s blood coagulation, inflammatory response, fat metabolism and other physiological processes (Lim et al., 2013). It has a positive regulatory effect on Rho protein signal transduction, cell phagocytosis and cell migration (Rattenholl et al., 2007). PAR-2 plays an important role in the transport of melanosomes from melanocytes to keratinocytes, especially in skin pigmentation caused by ultraviolet radiation and inflammation. After UV irradiation, PAR-2 can activate Rho signaling, enhance the dendritic formation of melanocytes, increase the phagocytic capacity of keratinocytes, increase the amount of melanosomes transported to keratinocytes, and aggravate skin pigmentation (Enomoto et al., 2011). After inflammatory stimulation, PAR-2 can induce the release of prostaglandin (PG) including PGE2 and PGF2α from keratinocytes. PG can enhance the dendrites of melanocytes and promote the transport of melanosome (G. Scott et al., 2004).

#### 3.2.3 MLPH - MyoVa - Rab27a

The human melanophilin (MLPH, also known as Slac2-a) gene is located on chromosome 2q37,3. The process of melanin accumulation in the periphery of vitro has been shown to be controlled by the unconventional myosin Va (MyoVa) (Mercer et al., 1991), the Ras-associated gtpase Rab27a (S. M. Wilson et al., 2000), and the Rab-effector MLPH (Matesic et al., 2001). The products of these three genes work together to anchor melanosomes to the actin cytoskeleton, thus facilitating their transport within the cell. MLPH binds with MyoVa at one end and Rab27a, which is itself targeted to the melanosomal membrane, at the other (Nascimento et al., 2003). They form a protein complex that has been shown to be essential for the capture and movement of melanosomes via the actin cytoskeleton (Hume et al., 2002; Provance et al., 2002; Strom et al., 2002; Wu et al., 2002). After actin-based melanosome transport, Rab27a interacts with Slp2-a, another Rab27-specific effector, and promotes the anchoring of melanosomes to the plasma membrane (Kuroda and Fukuda, 2004).

Mutations in the MLPH gene cause Griseli’s syndrome, which is characterized by abnormally light skin (hypopigmentation) and silver-gray hair. Different from Grieseli syndrome caused by other factors, MLPH gene mutations cause type 3 lesions. The biggest difference between this type of lesions and other Grieseli syndrome is that it does not involve abnormalities of the brain or immune system.

### 3.3 Melanin-related protein family

There are also many protein families involved in the formation and transport of melanosome, such as BLOC, Rab and Rho. BLOC protein family are mainly effect on the transfer of tyrosinase that control the formation of melanin. Rab protein family of proteins will affect the transfer of tyrosinase, the other part is the influence of transport of melanin body. And Rho protein family is mostly is by influencing the transport of melanin body to participate in the process of melanin expression.

#### 3.3.1 BLOC

BLOC (biogenesis of lysosome - related organelles complex, lysosome associated organelles biological complex). It is a multisubunit protein complex that is widely expressed in organisms and participates in the biosynthesis of special organelles of the endoplasmic lysosome system, such as melanosomes and platelets. BLOC is divided into three categories, BLOC-1, BLOC-2, and BLOC-3, which are closely related to HPS syndrome.

BLOC-1 is composed of eight subunits (pallidin, cappuccino, disbindin, snapin, muted, BLOS1, BLOS2, and BLOS3). disbindin mutations cause human HPS7 disease. BLOC-1 can sort TYRP1 and interact with PI4KII (Zhu et al., 2022), KIF13A and AP3 to transport TYRP1 from endosomal vesicles to melanosomes (Shakya et al., 2018; Thankachan and Setty, 2022).

BLOC-2 consists of three subunits (HPS3, HPS5, and HPS6), and mutations in HPS3, HPS5, and HPS6 cause HPS3, HPS5, and HPS6 disease, respectively (Wei et al., 2019). BLOC-2 can interact with Rab32/Rab38 to guide the endosomal recycling tubules to transport TYRP1 until melanosomes. In melanocytes deficient in BLOC-2, the number and length of endosomal tubules decreased, TYRP1 accumulated in endosomal vesicles, and the production of melanin decreased (Bultema et al., 2012).

BLOC-3 is composed of two subunits (HPS1 and HPS4), and mutations in HPS1 and HPS4 cause HPS1 and HPS4 diseases, respectively (De Jesus Rojas and Young, 2020). As a guanine nucleotide exchange factor of Rab32/Rab38, BLOC-3 deficiency directly affects the transport of melanin production related proteins (TYR, TYRP1, etc.) involved in Rab32/Rab38 (Gerondopoulos et al., 2012). For example, in HPS1 patients, there are phagocytotic vesicles near the melanosome, TYR and TYRP1 are engulfed by phagocytotic vesicles, leading to the block of melanin production (J. W. Smith et al., 2005).

#### 3.3.2 Rab

The Rab protein family is a member of the Ras superfamily of monomeric G-proteins, and approximately 70 Rab proteins have been identified in humans (Stenmark and Olkkonen, 2001). Rab proteins are important regulators of vesicle trafficking and can regulate many aspects of membrane trafficking, including vesicle formation, vesicle movement along actin and microtubule networks, vesicle budding, and membrane fusion (Diekmann et al., 2011). Among them, Rab7, Rab9a, Rab11, Rab17, Rab21, Rab27a, Rab32, Rab36, and Rab38 play important roles in the formation and transport of melanosomes.

Rab7 gene is located in human chromosome 3q21.3 (Kashuba et al., 1997). It is a key regulator of endolysosome transport and controls the maturation and migration of endolysosome (Bucci et al., 2000). Among the findings (Hida et al., 2011): Rab7 plays an important role in the transport of melanin production related proteins such as TYR, TYRP-1 and gp100 to melanosomes. RILP (Rab interacting lysosomal protein) is a downstream effector of Rab7, which links Rab7 function to the cytoskeleton and is related to the reverse transport of melanosomes (Agola et al., 2015).

The Rab9a gene is located in human chromosome Xp22.2 (Davies et al., 1997). It regulates the reverse transport of late vesicles to the Golgi apparatus. Rab9a can bind to RABEPK (Rab9 effector protein with Kelch motifs) (Díaz et al., 1997), M6PRBP1 (Mannose-6-phosphate receptor binding protein 1, M6PRBP1), and M6PRBP1 (mannose-6-phosphate receptor binding protein 1, M6PRBP1). Also known as TIP47 (Carroll et al., 2001), BLOC - 3 (Biogenesis of lysosome - related organelles complex 3) interaction (Kloer et al., 2010). For example, the interaction between Rab9a and BLOC 3 is involved in the formation of melanosome, and Rab9a gene mutation can cause HPS syndrome (A. H. Wei et al., 2013; Mahanty et al., 2016).

Rab11 is associated with both constitutive and regulatory secretory pathways and may be involved in protein transport, and Rab11 interacts with BLOC1 to participate in the trafficking of early vesicles during melanosome generation.

Rab17 (Beaumont et al., 2011) and Rab21 (Ohbayashi et al., 2012) are related to the formation of dendritic pseudopodia in melanocytes and affect the transport of melanosomes from melanocytes to keratinocytes. In addition, Rab17 expression may be regulated by MITF (Hoek et al., 2008).

Rab27a gene is located in human chromosome 15q21.3 (Tolmachova et al., 1999), which is a membrane-bound protein and may be involved in protein transport and small GTpase-mediated signal transduction. Rab27a protein plays an important role in melanosome transport. In melanocytes, MLPH could bind to MyoVa and Rab27a to form Rab27a-MLPH-MyoVa complex. This complex can transport melanosomes from the periphery of the nucleus to the end of dendrites (Kim et al., 2013). Mutations in any of the proteins in the complex will cause abnormal transport of melanosome and abnormal accumulation of mature melanosome around the nucleus (Kuroda et al., 2005). Mutations in MyoVa can cause Griscelli syndrome type 1 (GS1), which is characterized by characteristic hypopigmentation of skin and hair accompanied by neurological abnormal brain dysfunction (Ariffin et al., 2014). Rab27a mutation can cause Griscelli syndrome type 2 (GS2), the main symptoms of which are accompanied by immune abnormalities in addition to skin and hair hypopigination (Panigrahi et al., 2015). Mutations in MLPH cause Griscelli syndrome type 3 (GS3), which is characterized by hypopigmentation of the skin and hair (Van Gele et al., 2009; Bed’hom et al., 2012).

Rab32 and Rab38 are associated with melanosome formation (Bultema et al., 2012). It is involved in the transport of melanogenic proteins such as TYR and TYRP-1 to melanosome (Esposito et al., 2012). The study found that (Wasmeier et al., 2006): in Rab32 and Rab38 knockout melanocytes, TYR and TYRP-1 could not be transported to melsome and thus affect melanin production. In addition, mutations in the Rab32 gene of *Drosophila melanogaster* can cause hypopigoria (Wang et al., 2012).

Rab36 gene is located in human chromosome 22q11.23 (Mori et al., 1999), and similar to Rab7, it can interact with the effector protein RILP and participate in the reverse transport of melanosomes (Matsui et al., 2012).

#### 3.3.3 Rho

The Rho protein family is a member of the Ras superfamily of monomeric G-proteins, which belongs to the small gtpase proteins (∼21 kDa) and plays an important role in cellular functions such as organelle development, cytoskeletal dynamics, and cell motility (Ridley, 2015). At present, more than 20 members of the Rho protein family have been found in mammals, which are divided into 8 subfamilies, namely: Rac subfamily (Rac1, Rac2, Rac3, and RhoG), Cdc42 subfamily (Cdc42, TC10/RhoQ, and TCL/RhoJ), RhoUV subfamily (RhoV/Chp and RhoU/Wrch-1), Rho subfamily (RhoA, RhoB, and RhoC), Rnd subfamilies (Rnd1/Rho6, Rnd2/RhoN, and Rnd3/RhoE), RhoDF subfamilies (RhoD and RhoF/Rif), RhoBTB subfamilies (RhoBTB1 and RhoBTB2) and RhoH/TTF subfamilies(Boureux et al., 2007). Among them, Rac1, Cdc42, and RhoA play important roles in the dendrite, cytoskeleton, cell migration and melanosomes transport of melanocytes.

Rac1 can mediate the formation of lamellipodia, promote the extension of lamellipodia, prevent actin depolymerization, and play a positive regulatory role in the formation of cell dendrites (Tashiro and Yuste, 2004). In Rac1-deficient cells, cell morphology was shrunken, lamellipodia formation was blocked, and dendrite was not obvious (Guo et al., 2006).

Cdc42 is involved in dendritic growth, branching and branch stability. Cdc42 can mediate the formation of filopodia, promote filopodia elongation, prevent actin depolymerization, and play a positive regulatory role in cell dendrite formation (Scott et al., 2003). Studies have found that (Crawford et al., 1998), after Cdc42 deficiency in *Drosophila*, the dendritic morphology of neurons in the vertical system is changed, and the dendritic caliber and dendritic branch localization are defective. Cdc42 gene mutation can cause Wiskott-Aldrich syndrome, which is a recessive genetic disease, only affects males, and the membrane skeleton of lymphocytes and platelets in patients is abnormal (Nonoyama and Ochs, 2001).

RhoA can mediate the formation of stress fibers. RhoA binds to the downstream effector molecule ROCK, promotes the formation of stress fibers and focal adhesion, and promotes the polymerization of actin microfilaments, thereby shortening the dendrites and negatively regulating the formation of cell dendrites (Couvillon and Exton, 2006).

## 4 Mechanisms of action of popular whitening active agents

The study found that the dimension of country or region was positively correlated with the length of local skin brightness, and the difference gradually increased with the increase of age (Bae et al., 2016). Factors can be divided into external factors and internal factors. External factors such as ultraviolet radiation and drug consumption, and internal factors such as the body’s immune response and hormonal signals can affect melanin production in different ways (Rzepka et al., 2016). Intense ex-posure to UV radiation can cause a variety of conditions, ranging from mild sunburn and oxidative stress to DNA damage and skin cancer, and accelerate skin aging (Kong et al., 2016). We can based on some biological knowledge in the process of melanin production by melanocytes, to understand how some popular whitening products today, the products that really have whitening effect play their own value.

### 4.1 Disruption of tyrosinase activity: kojic acid

**FIGURE 3:**
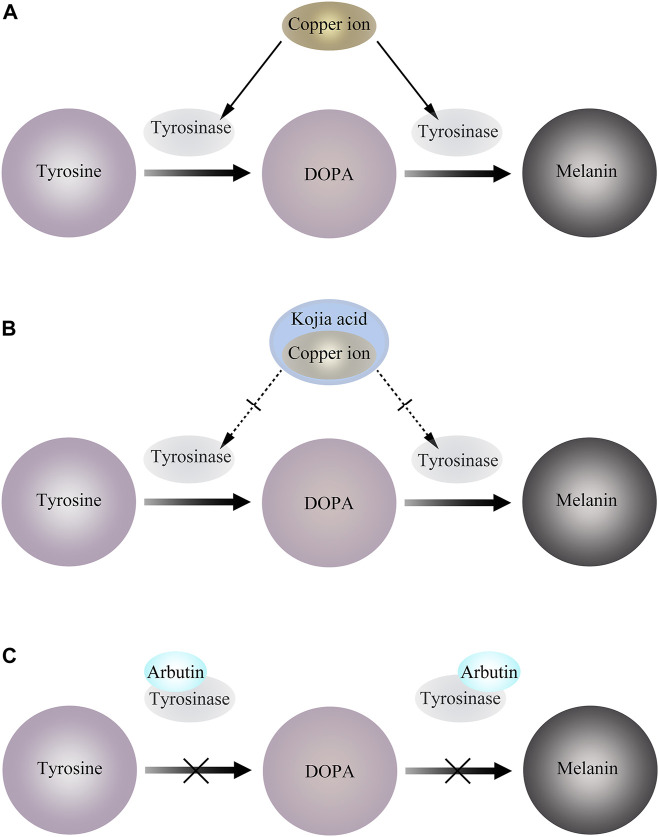
**(A)** Copper ions bind to tyrosinase and activate tyrosinase activity. **(B)** KA preemptively binds to copper ions by trapping copper ions, thereby inhibiting tyrosinase activity. **(C)** Arbutin binds to Emet and inhibits melanosome generation.

Kojic acid (KA) is a metabolite produced by fungi, and its derivatives exhibit biocompatibility, antibacterial, antiviral, anti-cancer, anti-spot, anti-parasitic, and insecticidal characteristics, among other things. Tyrosinase activity is activated and melanin formation is accelerated by UV radiation because copper ions, which are very active under UV light, can quickly attach to the copper active site of tyrosinase. To prevent copper ions from activating tyrosinase and suppressing tyrosinase activity, KA instead preemptively binds to copper ions by trapping copper ions. Due to its ability to reduce o-dopaquinone to L-Dopa and avoid melanin formation, KA has been extensively described in the literature as a potent anti-TYR agent (Saghaie et al., 2013) (Figure 3). Additionally, kojic acid is currently the most popular skin brightener and is frequently added to a wide range of skin care products so that the product has a particular impact. Yet kojic acid is not entirely beneficial and may have side effects like erythema, dermatitis, and hypersensitivity (T. S. Chang, 2009; Gillbro and Olsson, 2011).The available human sensitization data supported the safety of kojic acid at a use concentration of 2% in leave-on cosmetics (Burnett et al., 2010).

### 4.2 Preempt tyrosinase binding sites: arbutin

Arbutin is a natural active substance extracted from plants. Its biological structure is similar to hydroquinone, and its damage to the integrity of melanosomes is less than that of hydroquinone. At present, the common arbutin include α-arbutin, β-arbutin and deoxyarbutin. And the damage to the integrity of melanosomes was less than that of hydroquinone (Miao et al., 2016).

It has been reported that arbutin can be used as a substrate of TYR. In the presence of a certain amount of L-DOPA as a cofactor, arbutin is oxidized by mushroom TYR to 3,4-dihydroxyphenyl-O-beta-D-glucopyranoside (Nihei and Kubo, 2003), and can also inactivate TYR by binding to Emet in the absence of L-DOPA (Hori et al., 2004). This leads to interference of melanocytes, thereby blocking melanin production (Figure 3).

### 4.3 Inhibits the transfer of the generated melanin to the epidermis: niacinamide

**FIGURE 4:**
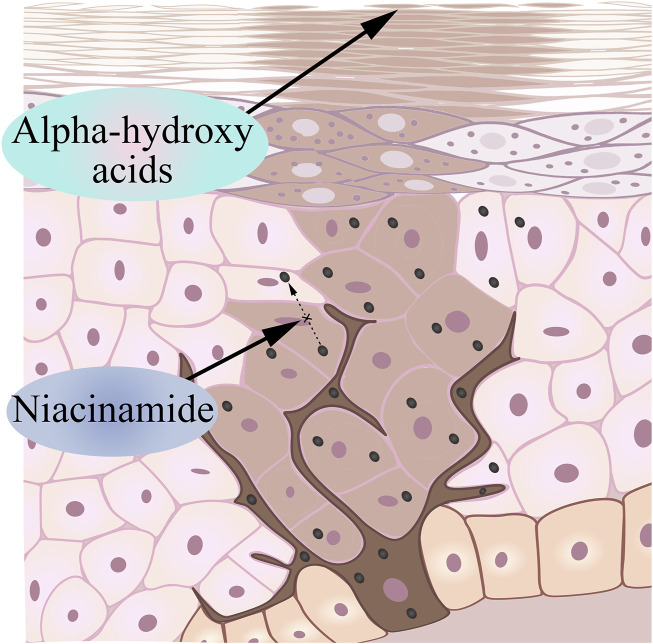
Nicotinamide inhibits melanosome transport and AHA accelerates keratinocyte shedding.

Nicotinamide is a bioactive form of niacin (vitamin B3) that has been shown to reduce pigmentation based on existing research (Russomanno et al., 2023). Human skin naturally undergoes the Maillard reaction as we age, which produces cross-linked proteins that give aging skin a yellowish color (Wolff et al., 1991; Bissett et al., 2004). Nicotinamide keeps melanin in melanocytes by interfering with the transport of melanosomes, causing melanocytes to no longer produce melanin, and the melanin that has been produced cannot reach keratinocytes (Figure 4). Interestingly, in addition to interfering with the transport of melanin bodies to produce whitening effects, the current research progress has proved that niacinamide can enhance the repair of UV-mediated DNA damage in keratinocytes, reduce UV-mediated inflammation, and prevent UV-induced immunosuppression (Rolfe, 2014; Snaidr et al., 2019).

Unfortunately, inhibition of melanin transfer by nicotinamide is reversible, and skin darkening occurs after cessation of nicotinamide.

### 4.4 Accelerate the degradation of melanin in the stratum corneum: alpha-hydroxy acids

Alpha-hydroxyacids (AHA) is an organic acid extracted from fruits, including grape acid, malic acid, citrus acid, etc., (Yu and Van Scott, 2002). Many AHAs are intermediate products or end metabolites in carbohydrate metabolism (Kai et al., 2019). Based on its properties, glycolic acid can chemically reduce the viscosity of glial cells in the epidermal layer, accelerate the exfoliation of keratinocytes, rapidly remove aging keratinocytes, and make melanin escape with glial cells (Sunder, 2019) (Figure 4). Due to the small molecules and the strong permeability, the appropriate dose of alpha hydroxy acids can act on the dermis, so it may stimulate keratinocytes and fibroblasts to produce various cytokines, adhesion molecules and growth factors, thereby improving and restoring the aging skin and achieving the whitening effect (Huang et al., 2022).

### 4.5 Reduce the effect of UV on melanin formation

In the past decade, the most popular category of cosmetics should be sunscreen products, the impact of ultraviolet (UV) on the skin is recognized to have immediate and delayed effects, tanning, sunburn, can be triggered in the absence of adequate UV protection, over time, the skin will change. Commonly used sunscreen products are divided into physical sunscreen and chemical sunscreen (Cole et al., 2023). Two physical sunscreens that are often added to sunscreens are titanium dioxide and zinc oxide, both of which have a strong anti-blue light effect (Bernstein et al., 2021). Titanium dioxide has high refractive properties and high photoactivity and is able to reflect UV light (Bernstein et al., 2021). Zinc oxide is an N-type semiconductor, and the electrons on the valence band can accept the energy transition in ultraviolet light, which is the principle of their absorption of ultraviolet light. Chemical sun protection is mainly through the absorption of ultraviolet rays, reduce ultraviolet rays into the epidermal layer to achieve the effect of sun protection, commonly used chemical sun protection are: diphenyl ketone, ethyl hexyl salicylate, Humosalate, xisoxamate and phenyl diphenyl imidazole tetraxanthate disodium, etc.

## 5 Conclusion and future expectations

In this paper, the research progress of related proteins involved in melanin synthesis is reviewed. From the origin of melanin, the proteins involved in the production and transport of melanin and how they affect the formation of melanin are described. TYR is a key enzyme in the process of melanin formation, which can directly affect the production of melanin. MITF, PKC, HNF-1α, SOX10, PAX3, p53, MC1R, OA1, Mlph, and PAR-2 related to it are all proteins related to the production of melanin. These proteins directly or indirectly control melanin production by affecting TYR. OA1, Mlph, and PAR-2 control the expression of melanin on the skin surface by affecting the transport of melanin bodies. In addition, this paper briefly summarizes several active ingredients with whitening effect on the market, and introduces their principle of blocking melanin production.

At present, the research on melanin is no longer on the surface. Relevant studies have proved that neural stem cell-derived CM can be used as a new material in cosmetics, and NSC-CM can be used as a core technology to meet the pursuit of fair skin in women (Hwang and Hong, 2017). And the research on melanin has been deeply applied to the medical field, such as using melanin as a probe in magnetic resonance imaging, antioxidant therapy, photothermal therapy, chemotherapy and therapeutics, tissue scaffold and sealing material supplement, and other PDAM applications (Solano, 2017). There are many diseases known to be related to melanin, such as albinism, melanoma, and various lesions caused by mutations in the genes related to melanin forming proteins mentioned in the text. In order to elucidate the interactions between a variety of transporters and proteins and ions, perhaps in addition to biological functions and physiological roles, we should incorporate the interactomics, which may better explain the function of proteins in the human body. Based on the published literature so far, the function and characteristics of melanosome-related transporters need to be further studied.

## References

[B1] Agola J. O. Sivalingam D. Cimino D. F. Simons P. C. Buranda T. Sklar L. A. (2015). Quantitative bead-based flow cytometry for assaying Rab7 GTPase interaction with the Rab-interacting lysosomal protein (RILP) effector protein. Methods Mol. Biol. 1298, 331–354. 10.1007/978-1-4939-2569-8_28 25800855 PMC6033261

[B2] Alkobtawi M. Ray H. Barriga E. H. Moreno M. Kerney R. Monsoro-Burq A. H. (2018). Characterization of Pax3 and Sox10 transgenic *Xenopus laevis* embryos as tools to study neural crest development. Dev. Biol. 444 (Suppl. 1), S202–S208. 10.1016/j.ydbio.2018.02.020 29522707 PMC6453020

[B3] Ando H. Niki Y. Ito M. Akiyama K. Matsui M. S. Yarosh D. B. (2012). Melanosomes are transferred from melanocytes to keratinocytes through the processes of packaging, release, uptake, and dispersion. J. Invest. Dermatol. 132, 1222–1229. 10.1038/jid.2011.413 22189785

[B4] Ando H. Niki Y. Yoshida M. Ito M. Akiyama K. Kim J. H. (2011). Involvement of pigment globules containing multiple melanosomes in the transfer of melanosomes from melanocytes to keratinocytes. Cell Logist. 1, 12–20. 10.4161/cl.1.1.13638 21686100 PMC3109459

[B5] Ariffin H. Geikowski A. Chin T. F. Chau D. Arshad A. Abu Bakar K. (2014). Griscelli syndrome. Med. J. Malays. 69, 193–194. 25500851

[B6] Bae S. H. Park J. J. Song E. J. Lee J. A. Byun K. S. Kim N. S. (2016). The comparison of the melanin content and UV exposure affecting aging process: seven countries in Asia. J. Cosmet. Dermatol 15, 335–342. 10.1111/jocd.12234 27369004

[B7] Barton D. E. Kwon B. S. Francke U. (1988). Human tyrosinase gene, mapped to chromosome 11 (q14----q21), defines second region of homology with mouse chromosome 7. Genomics 3, 17–24. 10.1016/0888-7543(88)90153-x 3146546

[B8] Baxter L. L. Hou L. Loftus S. K. Pavan W. J. (2004). Spotlight on spotted mice: a review of white spotting mouse mutants and associated human pigmentation disorders. Pigment. Cell Res. 17, 215–224. 10.1111/j.1600-0749.2004.00147.x 15140066

[B9] Beaumont K. A. Hamilton N. A. Moores M. T. Brown D. L. Ohbayashi N. Cairncross O. (2011). The recycling endosome protein Rab17 regulates melanocytic filopodia formation and melanosome trafficking. Traffic 12, 627–643. 10.1111/j.1600-0854.2011.01172.x 21291502

[B10] Bed’hom B. Vaez M. Coville J. L. Gourichon D. Chastel O. Follett S. (2012). The lavender plumage colour in Japanese quail is associated with a complex mutation in the region of MLPH that is related to differences in growth, feed consumption and body temperature. BMC Genomics 13, 442. 10.1186/1471-2164-13-442 22937744 PMC3484014

[B11] Bento-Lopes L. Cabaço L. C. Charneca J. Neto M. V. Seabra M. C. Barral D. C. (2023). Melanin's journey from melanocytes to keratinocytes: uncovering the molecular mechanisms of melanin transfer and processing. Int. J. Mol. Sci. 24, 11289. 10.3390/ijms241411289 37511054 PMC10379423

[B12] Bernstein E. F. Sarkas H. W. Boland P. (2021). Iron oxides in novel skin care formulations attenuate blue light for enhanced protection against skin damage. J. Cosmet. Dermatol 20, 532–537. 10.1111/jocd.13803 33210401 PMC7894303

[B13] Bertolotto C. Buscà R. Abbe P. Bille K. Aberdam E. Ortonne J. P. (1998). Different cis-acting elements are involved in the regulation of TRP1 and TRP2 promoter activities by cyclic AMP: pivotal role of M boxes (GTCATGTGCT) and of microphthalmia. Mol. Cell Biol. 18, 694–702. 10.1128/mcb.18.2.694 9447965 PMC108780

[B14] Bissett D. L. Miyamoto K. Sun P. Li J. Berge C. A. (2004). Topical niacinamide reduces yellowing, wrinkling, red blotchiness, and hyperpigmented spots in aging facial skin. Int. J. Cosmet. Sci. 26, 231–238. 10.1111/j.1467-2494.2004.00228.x 18492135

[B15] Bondurand N. Pingault V. Goerich D. E. Lemort N. Sock E. Le Caignec C. (2000). Interaction among SOX10, PAX3 and MITF, three genes altered in Waardenburg syndrome. Hum. Mol. Genet. 9, 1907–1917. 10.1093/hmg/9.13.1907 10942418

[B16] Bondurand N. Sham M. H. (2013). The role of SOX10 during enteric nervous system development. Dev. Biol. 382, 330–343. 10.1016/j.ydbio.2013.04.024 23644063

[B17] Bouchard B. Del Marmol V. Jackson I. J. Cherif D. Dubertret L. (1994). Molecular characterization of a human tyrosinase-related-protein-2 cDNA. Patterns of expression in melanocytic cells. Eur. J. Biochem. 219, 127–134. 10.1111/j.1432-1033.1994.tb19922.x 8306979

[B18] Boureux A. Vignal E. Faure S. Fort P. (2007). Evolution of the Rho family of ras-like GTPases in eukaryotes. Mol. Biol. Evol. 24, 203–216. 10.1093/molbev/msl145 17035353 PMC2665304

[B19] Box N. F. Wyeth J. R. Mayne C. J. O'Gorman L. E. Martin N. G. Sturm R. A. (1998). Complete sequence and polymorphism study of the human TYRP1 gene encoding tyrosinase-related protein 1. Mamm. Genome 9, 50–53. 10.1007/s003359900678 9434945

[B20] Britsch S. Goerich D. E. Riethmacher D. Peirano R. I. Rossner M. Nave K. A. (2001). The transcription factor Sox10 is a key regulator of peripheral glial development. Genes Dev. 15, 66–78. 10.1101/gad.186601 11156606 PMC312607

[B21] Bronner M. E. LeDouarin N. M. (2012). Development and evolution of the neural crest: an overview. Dev. Biol. 366, 2–9. 10.1016/j.ydbio.2011.12.042 22230617 PMC3351559

[B22] Bucci C. Thomsen P. Nicoziani P. McCarthy J. van Deurs B. (2000). Rab7: a key to lysosome biogenesis. Mol. Biol. Cell 11, 467–480. 10.1091/mbc.11.2.467 10679007 PMC14786

[B23] Bultema J. J. Ambrosio A. L. Burek C. L. Di Pietro S. M. (2012). BLOC-2, AP-3, and AP-1 proteins function in concert with Rab38 and Rab32 proteins to mediate protein trafficking to lysosome-related organelles. J. Biol. Chem. 287, 19550–19563. 10.1074/jbc.M112.351908 22511774 PMC3365991

[B24] Burnett C. L. Bergfeld W. F. Belsito D. V. Hill R. A. Klaassen C. D. Liebler D. C. (2010). Final report of the safety assessment of Kojic acid as used in cosmetics. Int. J. Toxicol. 29, 244S–273S. 10.1177/1091581810385956 21164073

[B25] Cao Y. Wang C. (2000). The COOH-terminal transactivation domain plays a key role in regulating the *in vitro* and *in vivo* function of Pax3 homeodomain. J. Biol. Chem. 275, 9854–9862. 10.1074/jbc.275.13.9854 10734141

[B26] Carroll K. S. Hanna J. Simon I. Krise J. Barbero P. Pfeffer S. R. (2001). Role of Rab9 GTPase in facilitating receptor recruitment by TIP47. Science 292, 1373–1376. 10.1126/science.1056791 11359012

[B27] Castejón-Griñán M. Herraiz C. Olivares C. Jiménez-Cervantes C. García-Borrón J. C. (2018). cAMP-independent non-pigmentary actions of variant melanocortin 1 receptor: AKT-mediated activation of protective responses to oxidative DNA damage. Oncogene 37, 3631–3646. 10.1038/s41388-018-0216-1 29622793

[B28] Catania A. Gatti S. Colombo G. Lipton J. M. (2004). Targeting melanocortin receptors as a novel strategy to control inflammation. Pharmacol. Rev. 56, 1–29. 10.1124/pr.56.1.1 15001661

[B29] Cerdan D. Redziniak G. Bourgeois C. A. Monsigny M. Kieda C. (1992). C32 human melanoma cell endogenous lectins: characterization and implication in vesicle-mediated melanin transfer to keratinocytes. Exp. Cell Res. 203, 164–173. 10.1016/0014-4827(92)90052-a 1426039

[B30] Chang T. Hashimoto K. Bawle E. V. (1993). Spontaneous contraction of leukodermic patches in Waardenburg syndrome. J. Dermatol 20, 707–711. 10.1111/j.1346-8138.1993.tb01368.x 8300941

[B31] Chang T. S. (2009). An updated review of tyrosinase inhibitors. Int. J. Mol. Sci. 10, 2440–2475. 10.3390/ijms10062440 19582213 PMC2705500

[B32] Cheli Y. Ohanna M. Ballotti R. Bertolotto C. (2010). Fifteen-year quest for microphthalmia-associated transcription factor target genes. Pigment. Cell Melanoma Res. 23, 27–40. 10.1111/j.1755-148X.2009.00653.x 19995375

[B33] Chen S. Zhu B. Yin C. Liu W. Han C. Chen B. (2017). Palmitoylation-dependent activation of MC1R prevents melanomagenesis. Nature 549, 399–403. 10.1038/nature23887 28869973 PMC5902815

[B34] Chhajlani V. Wikberg J. E. (1992). Molecular cloning and expression of the human melanocyte stimulating hormone receptor cDNA. FEBS Lett. 309, 417–420. 10.1016/0014-5793(92)80820-7 1516719

[B35] Cole Y. Ilyas A. M. Ilyas E. N. (2023). Availability of adequate photoprotection for skin of color. Cureus 15, e42794. 10.7759/cureus.42794 37664385 PMC10470041

[B36] Correia M. S. Moreiras H. Pereira F. J. C. Neto M. V. Festas T. C. Tarafder A. K. (2018). Melanin transferred to keratinocytes resides in nondegradative endocytic compartments. J. Invest. Dermatol 138, 637–646. 10.1016/j.jid.2017.09.042 29074272

[B37] Costa R. H. Kalinichenko V. V. Holterman A. X. L. Wang X. (2003). Transcription factors in liver development, differentiation, and regeneration. Hepatology 38, 1331–1347. 10.1016/j.hep.2003.09.034 14647040

[B38] Couvillon A. D. Exton J. H. (2006). Role of heterotrimeric G-proteins in lysophosphatidic acid-mediated neurite retraction by RhoA-dependent and -independent mechanisms in N1E-115 cells. Cell Signal 18, 715–728. 10.1016/j.cellsig.2005.06.012 16122906

[B39] Crawford J. M. Harden N. Leung T. Lim L. Kiehart D. P. (1998). Cellularization in *Drosophila melanogaster* is disrupted by the inhibition of rho activity and the activation of Cdc42 function. Dev. Biol. 204, 151–164. 10.1006/dbio.1998.9061 9851849

[B40] Cui R. Widlund H. R. Feige E. Lin J. Y. Wilensky D. L. Igras V. E. (2007). Central role of p53 in the suntan response and pathologic hyperpigmentation. Cell 128, 853–864. 10.1016/j.cell.2006.12.045 17350573

[B41] Davies J. P. Cotter P. D. Ioannou Y. A. (1997). Cloning and mapping of human Rab7 and Rab9 cDNA sequences and identification of a Rab9 pseudogene. Genomics 41, 131–134. 10.1006/geno.1997.4644 9126495

[B42] DeForest N. Kavitha B. Hu S. Isaac R. Krohn L. Wang M. (2023). Human gain-of-function variants in HNF1A confer protection from diabetes but independently increase hepatic secretion of atherogenic lipoproteins. Cell Genom 3, 100339. 10.1016/j.xgen.2023.100339 37492105 PMC10363808

[B43] De Jesus Rojas W. Young L. R. (2020). Hermansky-pudlak syndrome. Semin. Respir. Crit. Care Med. 41, 238–246. 10.1055/s-0040-1708088 32279294

[B44] Díaz E. Schimmöller F. Pfeffer S. R. (1997). A novel Rab9 effector required for endosome-to-TGN transport. J. Cell Biol. 138, 283–290. 10.1083/jcb.138.2.283 9230071 PMC2138197

[B45] Diekmann Y. Seixas E. Gouw M. Tavares-Cadete F. Seabra M. C. Pereira-Leal J. B. (2011). Thousands of rab GTPases for the cell biologist. PLoS Comput. Biol. 7, e1002217. 10.1371/journal.pcbi.1002217 22022256 PMC3192815

[B46] Donatien P. D. Hunt G. Pieron C. Lunec J. Taïeb A. Thody A. J. (1992). The expression of functional MSH receptors on cultured human melanocytes. Arch. Dermatol Res. 284, 424–426. 10.1007/bf00372074 1337693

[B47] Dupin E. Le Douarin N. M. (2003). Development of melanocyte precursors from the vertebrate neural crest. Oncogene 22, 3016–3023. 10.1038/sj.onc.1206460 12789276

[B48] Enomoto A. Yoshihisa Y. Yamakoshi T. Ur Rehman M. Norisugi O. Hara H. (2011). UV-B radiation induces macrophage migration inhibitory factor-mediated melanogenesis through activation of protease-activated receptor-2 and stem cell factor in keratinocytes. Am. J. Pathol. 178, 679–687. 10.1016/j.ajpath.2010.10.021 21281800 PMC3069914

[B49] Esposito R. D'Aniello S. Squarzoni P. Pezzotti M. R. Ristoratore F. Spagnuolo A. (2012). New insights into the evolution of metazoan tyrosinase gene family. PLoS One 7, e35731. 10.1371/journal.pone.0035731 22536431 PMC3334994

[B50] Fridman J. S. Lowe S. W. (2003). Control of apoptosis by p53. Oncogene 22, 9030–9040. 10.1038/sj.onc.1207116 14663481

[B51] Gantz I. Yamada T. Tashiro T. Konda Y. Shimoto Y. Miwa H. (1994). Mapping of the gene encoding the melanocortin-1 (alpha-melanocyte stimulating hormone) receptor (MC1R) to human chromosome 16q24.3 by Fluorescence *in situ* hybridization. Genomics 19, 394–395. 10.1006/geno.1994.1080 8188275

[B52] García-Borrón J. C. Sánchez-Laorden B. L. Jiménez-Cervantes C. (2005). Melanocortin-1 receptor structure and functional regulation. Pigment. Cell Res. 18, 393–410. 10.1111/j.1600-0749.2005.00278.x 16280005

[B53] Gerondopoulos A. Langemeyer L. Liang J. R. Linford A. Barr F. A. (2012). BLOC-3 mutated in Hermansky-Pudlak syndrome is a Rab32/38 guanine nucleotide exchange factor. Curr. Biol. 22, 2135–2139. 10.1016/j.cub.2012.09.020 23084991 PMC3502862

[B54] Gether U. (2000). Uncovering molecular mechanisms involved in activation of G protein-coupled receptors. Endocr. Rev. 21, 90–113. 10.1210/edrv.21.1.0390 10696571

[B55] Ghanem G. E. Comunale G. Libert A. Vercammen-Grandjean A. Lejeune F. J. (1988). Evidence for alpha-melanocyte-stimulating hormone (alpha-MSH) receptors on human malignant melanoma cells. Int. J. Cancer 41, 248–255. 10.1002/ijc.2910410216 2828246

[B56] Gillbro J. M. Olsson M. J. (2011). The melanogenesis and mechanisms of skin-lightening agents--existing and new approaches. Int. J. Cosmet. Sci. 33, 210–221. 10.1111/j.1468-2494.2010.00616.x 21265866

[B57] Goding C. R. Arnheiter H. (2019). MITF-the first 25 years. Genes Dev. 33, 983–1007. 10.1101/gad.324657.119 31123060 PMC6672050

[B58] Gross L. (2008). A molecular link between albinism and visual deficits. PLoS Biol. 6, e248. 10.1371/journal.pbio.0060248 20076724 PMC2553851

[B59] Guida S. Guida G. Goding C. R. (2022). MC1R functions, expression, and implications for targeted therapy. J. Invest. Dermatol. 142, 293–302.e1. 10.1016/j.jid.2021.06.018 34362555

[B60] Guo F. Debidda M. Yang L. Williams D. A. Zheng Y. (2006). Genetic deletion of Rac1 GTPase reveals its critical role in actin stress fiber formation and focal adhesion complex assembly. J. Biol. Chem. 281, 18652–18659. 10.1074/jbc.M603508200 16698790

[B61] Hachiya A. Kobayashi A. Yoshida Y. Kitahara T. Takema Y. Imokawa G. (2004). Biphasic expression of two paracrine melanogenic cytokines, stem cell factor and endothelin-1, in ultraviolet B-induced human melanogenesis. Am. J. Pathol. 165, 2099–2109. 10.1016/s0002-9440(10)63260-9 15579452 PMC1618730

[B62] Hellström A. R. Watt B. Fard S. S. Tenza D. Mannström P. Narfström K. (2011). Inactivation of Pmel alters melanosome shape but has only a subtle effect on visible pigmentation. PLoS Genet. 7, e1002285. 10.1371/journal.pgen.1002285 21949658 PMC3174228

[B63] Hershey C. L. Fisher D. E. (2005). Genomic analysis of the Microphthalmia locus and identification of the MITF-J/Mitf-J isoform. Gene 347, 73–82. 10.1016/j.gene.2004.12.002 15715979

[B64] Hida T. Sohma H. Kokai Y. Kawakami A. Hirosaki K. Okura M. (2011). Rab7 is a critical mediator in vesicular transport of tyrosinase-related protein 1 in melanocytes. J. Dermatol 38, 432–441. 10.1111/j.1346-8138.2010.01004.x 21352276

[B65] Hodgkinson C. A. Nakayama A. Li H. Swenson L. B. Opdecamp K. Asher J. H. (1998). Mutation at the anophthalmic white locus in Syrian hamsters: haploinsufficiency in the Mitf gene mimics human Waardenburg syndrome type 2. Hum. Mol. Genet. 7, 703–708. 10.1093/hmg/7.4.703 9499424

[B66] Hoek K. S. Schlegel N. C. Eichhoff O. M. Widmer D. S. Praetorius C. Einarsson S. O. (2008). Novel MITF targets identified using a two-step DNA microarray strategy. Pigment. Cell Melanoma Res. 21, 665–676. 10.1111/j.1755-148X.2008.00505.x 19067971

[B67] Hollenbach A. D. McPherson C. J. Lagutina I. Grosveld G. (2002). The EF-hand calcium-binding protein calmyrin inhibits the transcriptional and DNA-binding activity of Pax3. Biochim. Biophys. Acta 1574, 321–328. 10.1016/s0167-4781(02)00230-0 11997098

[B68] Hong C. S. Saint-Jeannet J. P. (2005). Sox proteins and neural crest development. Semin. Cell Dev. Biol. 16, 694–703. 10.1016/j.semcdb.2005.06.005 16039883

[B69] Hori I. Nihei K. i. Kubo I. (2004). Structural criteria for depigmenting mechanism of arbutin. Phytother. Res. 18, 475–479. 10.1002/ptr.1456 15287073

[B70] Hou L. Pavan W. J. (2008). Transcriptional and signaling regulation in neural crest stem cell-derived melanocyte development: do all roads lead to Mitf? Cell Res. 18, 1163–1176. 10.1038/cr.2008.303 19002157

[B71] Huang Q. Chen D. Pan S. Hu M. Wang P. Wang H. (2022). Efficacy of alpha hydroxy acid combined with intense pulsed light in the treatment of acne vulgaris: a meta-analysis. J. Cosmet. Dermatol 21, 5642–5650. 10.1111/jocd.15186 35763391

[B72] Hume A. N. Collinson L. M. Hopkins C. R. Strom M. Barral D. C. Bossi G. (2002). The leaden gene product is required with Rab27a to recruit myosin Va to melanosomes in melanocytes. Traffic 3, 193–202. 10.1034/j.1600-0854.2002.030305.x 11886590

[B73] Hurbain I. Romao M. Sextius P. Bourreau E. Marchal C. Bernerd F. (2018). Melanosome distribution in keratinocytes in different skin types: melanosome clusters are not degradative organelles. J. Invest. Dermatol. 138, 647–656. 10.1016/j.jid.2017.09.039 29054596

[B74] Hwang I. Hong S. (2017). Neural stem cells and its derivatives as a new material for melanin inhibition. Int. J. Mol. Sci. 19, 36. 10.3390/ijms19010036 29271951 PMC5795986

[B75] Hwang Y. S. Kim Y. J. Kim M. O. Kang M. Oh S. W. Nho Y. H. (2017). Cannabidiol upregulates melanogenesis through CB1 dependent pathway by activating p38 MAPK and p42/44 MAPK. Chem. Biol. Interact. 273, 107–114. 10.1016/j.cbi.2017.06.005 28601556

[B76] Imokawa G. Kobayashi T. Miyagishi M. Higashi K. Yada Y. (1997). The role of endothelin-1 in epidermal hyperpigmentation and signaling mechanisms of mitogenesis and melanogenesis. Pigment. Cell Res. 10, 218–228. 10.1111/j.1600-0749.1997.tb00488.x 9263329

[B77] Jacobs-Cohen R. J. Wade P. R. Gershon M. D. (2002). Suppression of the melanogenic potential of migrating neural crest-derived cells by the branchial arches. Anat. Rec. 268, 16–26. 10.1002/ar.10132 12209561

[B78] Jiménez M. Kameyama K. Maloy W. L. Tomita Y. Hearing V. J. (1988). Mammalian tyrosinase: biosynthesis, processing, and modulation by melanocyte-stimulating hormone. Proc. Natl. Acad. Sci. U. S. A. 85, 3830–3834. 10.1073/pnas.85.11.3830 3131764 PMC280313

[B79] Kai F. Drain A. P. Weaver V. M. (2019). The extracellular matrix modulates the metastatic journey. Dev. Cell 49, 332–346. 10.1016/j.devcel.2019.03.026 31063753 PMC6527347

[B80] Kashuba V. I. Gizatullin R. Z. Protopopov A. I. Allikmets R. Korolev S. Li J. (1997). NotI linking/jumping clones of human chromosome 3: mapping of the TFRC, RAB7 and HAUSP genes to regions rearranged in leukemia and deleted in solid tumors. FEBS Lett. 419, 181–185. 10.1016/s0014-5793(97)01449-x 9428630

[B81] Kastan M. B. Canman C. E. Leonard C. J. (1995). P53, cell cycle control and apoptosis: implications for cancer. Cancer Metastasis Rev. 14, 3–15. 10.1007/bf00690207 7606818

[B82] Kastenhuber E. R. Lowe S. W. (2017). Putting p53 in context. Cell 170, 1062–1078. 10.1016/j.cell.2017.08.028 28886379 PMC5743327

[B83] Kim B. Lee J. Y. Lee H. Y. Nam K. Y. Park J. Lee S. M. (2013). Hesperidin suppresses melanosome transport by blocking the interaction of Rab27A-melanophilin. Biomol. Ther. Seoul. 21, 343–348. 10.4062/biomolther.2013.032 24244821 PMC3825197

[B84] Kloer D. P. Rojas R. Ivan V. Moriyama K. van Vlijmen T. Murthy N. (2010). Assembly of the biogenesis of lysosome-related organelles complex-3 (BLOC-3) and its interaction with Rab9. J. Biol. Chem. 285, 7794–7804. 10.1074/jbc.M109.069088 20048159 PMC2844223

[B85] Kobayashi T. Urabe K. Winder A. Jiménez-Cervantes C. Imokawa G. Brewington T. (1994). Tyrosinase related protein 1 (TRP1) functions as a DHICA oxidase in melanin biosynthesis. Embo J. 13, 5818–5825. 10.1002/j.1460-2075.1994.tb06925.x 7813420 PMC395555

[B86] Kong L. Wang S. Wu X. Zuo F. Qin H. Wu J. (2016). Paeoniflorin attenuates ultraviolet B-induced apoptosis in human keratinocytes by inhibiting the ROS-p38-p53 pathway. Mol. Med. Rep. 13, 3553–3558. 10.3892/mmr.2016.4953 26936104

[B87] Kroumpouzos G. Urabe K. Kobayashi T. Sakai C. Hearing V. J. (1994). Functional analysis of the slaty gene product (TRP2) as dopachrome tautomerase and the effect of a point mutation on its catalytic function. Biochem. Biophys. Res. Commun. 202, 1060–1068. 10.1006/bbrc.1994.2036 8048919

[B88] Kumar C. M. Sathisha U. V. Dharmesh S. Rao A. G. A. Singh S. A. (2011). Interaction of sesamol (3,4-methylenedioxyphenol) with tyrosinase and its effect on melanin synthesis. Biochimie 93, 562–569. 10.1016/j.biochi.2010.11.014 21144881

[B89] Kumar S. Rao K. (2012). Waardenburg syndrome: a rare genetic disorder, a report of two cases. Indian J. Hum. Genet. 18, 254–255. 10.4103/0971-6866.100804 23162308 PMC3491306

[B90] Kuroda T. S. Fukuda M. (2004). Rab27A-binding protein Slp2-a is required for peripheral melanosome distribution and elongated cell shape in melanocytes. Nat. Cell Biol. 6, 1195–1203. 10.1038/ncb1197 15543135

[B91] Kuroda T. S. Itoh T. Fukuda M. (2005). Functional analysis of slac2-a/melanophilin as a linker protein between Rab27A and myosin Va in melanosome transport. Methods Enzymol. 403, 419–431. 10.1016/s0076-6879(05)03037-5 16473608

[B92] Lai X. Wichers H. J. Soler-Lopez M. Dijkstra B. W. (2018). Structure and function of human tyrosinase and tyrosinase-related proteins. Chemistry 24, 47–55. 10.1002/chem.201704410 29052256

[B93] Lam B. L. Fingert J. H. Shutt B. C. Singleton E. M. Merin L. M. Brown H. H. (1997). Clinical and molecular characterization of a family affected with X-linked ocular albinism (OA1). Ophthalmic Genet. 18, 175–184. 10.3109/13816819709041432 9457748

[B94] Lang D. Chen F. Milewski R. Lu M. M. Epstein J. A. (2000). Pax3 is required for enteric ganglia formation and functions with Sox10 to modulate expression of c-ret. J. Clin. Invest. 106, 963–971. 10.1172/jci10828 11032856 PMC314346

[B95] Lapierre-Landry M. Carroll J. Skala M. C. (2018). Imaging retinal melanin: a review of current technologies. J. Biol. Eng. 12, 29. 10.1186/s13036-018-0124-5 30534199 PMC6280494

[B96] Leonard L. J. Townsend D. King R. A. (1988). Function of dopachrome oxidoreductase and metal ions in dopachrome conversion in the eumelanin pathway. Biochemistry 27, 6156–6159. 10.1021/bi00416a049 3142518

[B97] Lim J. Iyer A. Liu L. Suen J. Y. Lohman R. J. Seow V. (2013). Diet-induced obesity, adipose inflammation, and metabolic dysfunction correlating with PAR2 expression are attenuated by PAR2 antagonism. Faseb J. 27, 4757–4767. 10.1096/fj.13-232702 23964081

[B98] Lopez V. M. Decatur C. L. Stamer W. D. Lynch R. M. McKay B. S. (2008). L-DOPA is an endogenous ligand for OA1. PLoS Biol. 6, e236. 10.1371/journal.pbio.0060236 18828673 PMC2553842

[B99] Ludwig A. Rehberg S. Wegner M. (2004). Melanocyte-specific expression of dopachrome tautomerase is dependent on synergistic gene activation by the Sox10 and Mitf transcription factors. FEBS Lett. 556, 236–244. 10.1016/s0014-5793(03)01446-7 14706856

[B100] Mahanty S. Ravichandran K. Chitirala P. Prabha J. Jani R. A. Setty S. R. G. (2016). Rab9A is required for delivery of cargo from recycling endosomes to melanosomes. Pigment. Cell Melanoma Res. 29, 43–59. 10.1111/pcmr.12434 26527546 PMC4690521

[B101] Matesic L. E. Yip R. Reuss A. E. Swing D. A. O'Sullivan T. N. Fletcher C. F. (2001). Mutations in Mlph, encoding a member of the Rab effector family, cause the melanosome transport defects observed in leaden mice. Proc. Natl. Acad. Sci. U. S. A. 98, 10238–10243. 10.1073/pnas.181336698 11504925 PMC56945

[B102] Matsui T. Ohbayashi N. Fukuda M. (2012). The Rab interacting lysosomal protein (RILP) homology domain functions as a novel effector domain for small GTPase Rab36: rab36 regulates retrograde melanosome transport in melanocytes. J. Biol. Chem. 287, 28619–28631. 10.1074/jbc.M112.370544 22740695 PMC3436592

[B103] Mercer J. A. Seperack P. K. Strobel M. C. Copeland N. G. Jenkins N. A. (1991). Novel myosin heavy chain encoded by murine dilute coat colour locus. Nature 349, 709–713. 10.1038/349709a0 1996138

[B104] Miao F. Shi Y. Fan Z. F. Jiang S. Xu S. Z. Lei T. C. (2016). Deoxyarbutin possesses a potent skin-lightening capacity with No discernible cytotoxicity against melanosomes. PLoS One 11, e0165338. 10.1371/journal.pone.0165338 27776184 PMC5077105

[B105] Mollaaghababa R. Pavan W. J. (2003). The importance of having your SOX on: role of SOX10 in the development of neural crest-derived melanocytes and glia. Oncogene 22, 3024–3034. 10.1038/sj.onc.1206442 12789277

[B106] Montero-Melendez T. (2015). ACTH: the forgotten therapy. Semin. Immunol. 27, 216–226. 10.1016/j.smim.2015.02.003 25726511

[B107] Montero-Melendez T. Boesen T. Jonassen T. E. N. (2022). Translational advances of melanocortin drugs: integrating biology, chemistry and genetics. Semin. Immunol. 59, 101603. 10.1016/j.smim.2022.101603 35341670

[B108] Moreiras H. Seabra M. C. Barral D. C. (2021). Melanin transfer in the epidermis: the pursuit of skin pigmentation control mechanisms. Int. J. Mol. Sci. 22, 4466. 10.3390/ijms22094466 33923362 PMC8123122

[B109] Mori T. Fukuda Y. Kuroda H. Matsumura T. Ota S. Sugimoto T. (1999). Cloning and characterization of a novel Rab-family gene, Rab36, within the region at 22q11.2 that is homozygously deleted in malignant rhabdoid tumors. Biochem. Biophys. Res. Commun. 254, 594–600. 10.1006/bbrc.1998.9968 9920784

[B110] Mountjoy K. G. Robbins L. S. Mortrud M. T. Cone R. D. (1992). The cloning of a family of genes that encode the melanocortin receptors. Science 257, 1248–1251. 10.1126/science.1325670 1325670

[B111] Murase D. Hachiya A. Amano Y. Ohuchi A. Kitahara T. Takema Y. (2009). The essential role of p53 in hyperpigmentation of the skin via regulation of paracrine melanogenic cytokine receptor signaling. J. Biol. Chem. 284, 4343–4353. 10.1074/jbc.M805570200 19098008

[B112] Nagatsu T. Nakashima A. Watanabe H. Ito S. Wakamatsu K. (2022). Neuromelanin in Parkinson's disease: tyrosine hydroxylase and tyrosinase. Int. J. Mol. Sci. 23, 4176. 10.3390/ijms23084176 35456994 PMC9029562

[B113] Nakayama A. Nguyen M. T. Chen C. C. Opdecamp K. Hodgkinson C. A. Arnheiter H. (1998). Mutations in microphthalmia, the mouse homolog of the human deafness gene MITF, affect neuroepithelial and neural crest-derived melanocytes differently. Mech. Dev. 70, 155–166. 10.1016/s0925-4773(97)00188-3 9510032

[B114] Nascimento A. A. Roland J. T. Gelfand V. I. (2003). Pigment cells: a model for the study of organelle transport. Annu. Rev. Cell Dev. Biol. 19, 469–491. 10.1146/annurev.cellbio.19.111401.092937 14570578

[B115] Nihei K. Kubo I. (2003). Identification of oxidation product of arbutin in mushroom tyrosinase assay system. Bioorg Med. Chem. Lett. 13, 2409–2412. 10.1016/s0960-894x(03)00395-0 12824045

[B116] Nilforoushzadeh M. A. Farshi S. Nouri M. Alavi S. Bayat Tork B. Jaffary F. (2022). Transplantation of autologous epidermal melanocyte-keratinocyte cells suspension for cell therapy of vitiligo: a clinical evaluation and biometric assessment. J. Cosmet. Dermatol 21, 7147–7152. 10.1111/jocd.15423 36208002

[B117] Noisa P. Raivio T. (2014). Neural crest cells: from developmental biology to clinical interventions. Birth Defects Res. C Embryo Today 102, 263–274. 10.1002/bdrc.21074 25226872

[B118] Nonoyama S. Ochs H. D. (2001). Wiskott-Aldrich syndrome. Curr. Allergy Asthma Rep. 1, 430–437. 10.1007/s11882-001-0028-0 11892069

[B119] Odom D. T. Zizlsperger N. Gordon D. B. Bell G. W. Rinaldi N. J. Murray H. L. (2004). Control of pancreas and liver gene expression by HNF transcription factors. Science 303, 1378–1381. 10.1126/science.1089769 14988562 PMC3012624

[B120] Oetting W. S. (2002). New insights into ocular albinism type 1 (OA1): mutations and polymorphisms of the OA1 gene. Hum. Mutat. 19, 85–92. 10.1002/humu.10034 11793467

[B121] Oetting W. S. King R. A. (1999). Molecular basis of albinism: mutations and polymorphisms of pigmentation genes associated with albinism. Hum. Mutat. 13, 99–115. 10.1002/(sici)1098-1004(1999)13:2<99::Aid-humu2>3.0.Co;2-c 10094567

[B122] Ohbayashi N. Yatsu A. Tamura K. Fukuda M. (2012). The Rab21-GEF activity of Varp, but not its Rab32/38 effector function, is required for dendrite formation in melanocytes. Mol. Biol. Cell 23, 669–678. 10.1091/mbc.E11-04-0324 22171327 PMC3279394

[B123] Okazaki K. Uzuka M. Morikawa F. Toda K. Seiji M. (1976). Transfer mechanism of melanosomes in epidermal cell culture. J. Invest. Dermatol 67, 541–547. 10.1111/1523-1747.ep12664554 787440

[B124] Panigrahi I. Suthar R. Rawat A. Behera B. (2015). Seizure as the presenting manifestation in Griscelli syndrome type 2. Pediatr. Neurol. 52, 535–538. 10.1016/j.pediatrneurol.2015.01.010 25801174

[B125] Pingault V. Girard M. Bondurand N. Dorkins H. Van Maldergem L. Mowat D. (2002). SOX10 mutations in chronic intestinal pseudo-obstruction suggest a complex physiopathological mechanism. Hum. Genet. 111, 198–206. 10.1007/s00439-002-0765-8 12189494

[B126] Potterf S. B. Furumura M. Dunn K. J. Arnheiter H. Pavan W. J. (2000). Transcription factor hierarchy in Waardenburg syndrome: regulation of MITF expression by SOX10 and PAX3. Hum. Genet. 107, 1–6. 10.1007/s004390000328 10982026

[B127] Poulat F. Girard F. Chevron M. P. Gozé C. Rebillard X. Calas B. (1995). Nuclear localization of the testis determining gene product SRY. J. Cell Biol. 128, 737–748. 10.1083/jcb.128.5.737 7876301 PMC2120386

[B128] Preising M. Op de Laak J. P. Lorenz B. (2001). Deletion in the OA1 gene in a family with congenital X linked nystagmus. Br. J. Ophthalmol. 85, 1098–1103. 10.1136/bjo.85.9.1098 11520764 PMC1724103

[B129] Provance D. W. James T. L. Mercer J. A. (2002). Melanophilin, the product of the leaden locus, is required for targeting of myosin-Va to melanosomes. Traffic 3, 124–132. 10.1034/j.1600-0854.2002.030205.x 11929602 PMC1351229

[B130] Rattenholl A. Seeliger S. Buddenkotte J. Schön M. Schön M. P. Ständer S. (2007). Proteinase-activated receptor-2 (PAR2): a tumor suppressor in skin carcinogenesis. J. Invest. Dermatol 127, 2245–2252. 10.1038/sj.jid.5700847 17476297

[B131] Ridley A. J. (2015). Rho GTPase signalling in cell migration. Curr. Opin. Cell Biol. 36, 103–112. 10.1016/j.ceb.2015.08.005 26363959 PMC4728192

[B132] Rinne P. Rami M. Nuutinen S. Santovito D. van der Vorst E. P. C. Guillamat-Prats R. (2017). Melanocortin 1 receptor signaling regulates cholesterol transport in macrophages. Circulation 136, 83–97. 10.1161/circulationaha.116.025889 28450348 PMC5518461

[B133] Roberts D. W. Newton R. A. Beaumont K. A. Helen Leonard J. Sturm R. A. (2006). Quantitative analysis of MC1R gene expression in human skin cell cultures. Pigment. Cell Res. 19, 76–89. 10.1111/j.1600-0749.2005.00286.x 16420249

[B134] Rolfe H. M. (2014). A review of nicotinamide: treatment of skin diseases and potential side effects. J. Cosmet. Dermatol 13, 324–328. 10.1111/jocd.12119 25399625

[B135] Russomanno K. Abdel Azim S. Patel V. A. (2023). Immunomodulators for non-melanoma skin cancers: updated perspectives. Clin. Cosmet. Investig. Dermatol 16, 1025–1045. 10.2147/ccid.S362171 PMC10122480 37095898

[B136] Rzepka Z. Buszman E. Beberok A. Wrześniok D. (2016). From tyrosine to melanin: signaling pathways and factors regulating melanogenesis. Postepy Hig. Med. Dosw 70, 695–708. 10.5604/17322693.1208033 27356601

[B137] Saghaie L. Pourfarzam M. Fassihi A. Sartippour B. (2013). Synthesis and tyrosinase inhibitory properties of some novel derivatives of kojic acid. Res. Pharm. Sci. 8, 233–242. 24082892 PMC3757588

[B138] Sallmann G. B. Bray P. J. Rogers S. Quince A. Cotton R. G. H. Carden S. M. (2006). Scanning the ocular albinism 1 (OA1) gene for polymorphisms in congenital nystagmus by DHPLC. Ophthalmic Genet. 27, 43–49. 10.1080/13816810600677834 16754205

[B139] Sánchez-Mejías A. Watanabe Y. M Fernández R. López-Alonso M. Antiñolo G. Bondurand N. (2010). Involvement of SOX10 in the pathogenesis of Hirschsprung disease: report of a truncating mutation in an isolated patient. J. Mol. Med. Berl. 88, 507–514. 10.1007/s00109-010-0592-7 20130826 PMC3235085

[B140] Sarangarajan R. Boissy R. E. (2001). Tyrp1 and oculocutaneous albinism type 3. Pigment. Cell Res. 14, 437–444. 10.1034/j.1600-0749.2001.140603.x 11775055

[B141] Schallreuter K. U. Kothari S. Chavan B. Spencer J. D. (2008). Regulation of melanogenesis-controversies and new concepts. Exp. Dermatol 17, 395–404. 10.1111/j.1600-0625.2007.00675.x 18177348

[B142] Schallreuter K. U. Kothari S. Hasse S. Kauser S. Lindsey N. J. Gibbons N. C. J. (2003). *In situ* and *in vitro* evidence for DCoH/HNF-1 alpha transcription of tyrosinase in human skin melanocytes. Biochem. Biophys. Res. Commun. 301, 610–616. 10.1016/s0006-291x(02)03076-0 12565907

[B143] Scott E. K. Reuter J. E. Luo L. (2003). Small GTPase Cdc42 is required for multiple aspects of dendritic morphogenesis. J. Neurosci. 23, 3118–3123. 10.1523/jneurosci.23-08-03118.2003 12716918 PMC6742332

[B144] Scott G. Leopardi S. Printup S. Madden B. C. (2002). Filopodia are conduits for melanosome transfer to keratinocytes. J. Cell Sci. 115, 1441–1451. 10.1242/jcs.115.7.1441 11896192

[B145] Scott G. Leopardi S. Printup S. Malhi N. Seiberg M. Lapoint R. (2004). Proteinase-activated receptor-2 stimulates prostaglandin production in keratinocytes: analysis of prostaglandin receptors on human melanocytes and effects of PGE2 and PGF2alpha on melanocyte dendricity. J. Invest. Dermatol 122, 1214–1224. 10.1111/j.0022-202X.2004.22516.x 15140225

[B146] Shakya S. Sharma P. Bhatt A. M. Jani R. A. Delevoye C. Setty S. R. (2018). Rab22A recruits BLOC-1 and BLOC-2 to promote the biogenesis of recycling endosomes. EMBO Rep. 19, e45918. 10.15252/embr.201845918 30404817 PMC6280653

[B147] Shen B. Samaraweera P. Rosenberg B. Orlow S. J. (2001). Ocular albinism type 1: more than meets the eye. Pigment. Cell Res. 14, 243–248. 10.1034/j.1600-0749.2001.140403.x 11549106

[B148] Shi J. Guo Y. Wang H. Xiao Y. Liu W. Lyu L. (2022). The ubiquitin-proteasome system in melanin metabolism. J. Cosmet. Dermatol 21, 6661–6668. 10.1111/jocd.15433 36207998

[B149] Shih D. Q. Bussen M. Sehayek E. Ananthanarayanan M. Shneider B. L. Suchy F. J. (2001). Hepatocyte nuclear factor-1alpha is an essential regulator of bile acid and plasma cholesterol metabolism. Nat. Genet. 27, 375–382. 10.1038/86871 11279518

[B150] Siegrist W. Solca F. Stutz S. Giuffrè L. Carrel S. Girard J. (1989). Characterization of receptors for alpha-melanocyte-stimulating hormone on human melanoma cells. Cancer Res. 49, 6352–6358. 2804981

[B151] Siegrist W. Stutz S. Eberle A. N. (1994). Homologous and heterologous regulation of alpha-melanocyte-stimulating hormone receptors in human and mouse melanoma cell lines. Cancer Res. 54, 2604–2610. 8168086

[B152] Singh K. Baker R. Sikkink S. K. Nizard C. Schnebert S. Kurfurst R. (2017). E-cadherin mediates ultraviolet radiation- and calcium-induced melanin transfer in human skin cells. Exp. Dermatol 26, 1125–1133. 10.1111/exd.13395 28636748

[B153] Smith J. W. Koshoffer A. Morris R. E. Boissy R. E. (2005). Membranous complexes characteristic of melanocytes derived from patients with Hermansky-Pudlak syndrome type 1 are macroautophagosomal entities of the lysosomal compartment. Pigment. Cell Res. 18, 417–426. 10.1111/j.1600-0749.2005.00265.x 16280007 PMC1635962

[B154] Smith S. D. Kelley P. M. Kenyon J. B. Hoover D. (2000). Tietz syndrome (hypopigmentation/deafness) caused by mutation of MITF. J. Med. Genet. 37, 446–448. 10.1136/jmg.37.6.446 10851256 PMC1734605

[B155] Snaidr V. A. Damian D. L. Halliday G. M. (2019). Nicotinamide for photoprotection and skin cancer chemoprevention: a review of efficacy and safety. Exp. Dermatol 28 (Suppl. 1), 15–22. 10.1111/exd.13819 30698874

[B156] Solano F. (2017). Melanin and melanin-related polymers as materials with biomedical and biotechnological applications-cuttlefish ink and mussel foot proteins as inspired biomolecules. Int. J. Mol. Sci. 18, 1561. 10.3390/ijms18071561 28718807 PMC5536049

[B157] Sommer L. (2011). Generation of melanocytes from neural crest cells. Pigment. Cell Melanoma Res. 24, 411–421. 10.1111/j.1755-148X.2011.00834.x 21310010

[B158] Staleva L. Orlow S. J. (2006). Ocular albinism 1 protein: trafficking and function when expressed in *Saccharomyces cerevisiae* . Exp. Eye Res. 82, 311–318. 10.1016/j.exer.2005.07.003 16154128

[B159] Stenmark H. Olkkonen V. M. (2001). The Rab GTPase family. Genome Biol. 2, Reviews3007. 10.1186/gb-2001-2-5-reviews3007 11387043 PMC138937

[B160] Strom M. Hume A. N. Tarafder A. K. Barkagianni E. Seabra M. C. (2002). A family of Rab27-binding proteins. Melanophilin links Rab27a and myosin Va function in melanosome transport. J. Biol. Chem. 277, 25423–25430. 10.1074/jbc.M202574200 11980908

[B161] Sunder S. (2019). Relevant topical skin care products for prevention and treatment of aging skin. Facial Plast. Surg. Clin. North Am. 27, 413–418. 10.1016/j.fsc.2019.04.007 31280856

[B162] Swift J. A. (1964). Transfer of melanin granules from melanocytes to the cortical cells of human hair. Nature 203, 976–977. 10.1038/203976b0 14203516

[B163] Swope V. B. Abdel-Malek Z. A. (2018). MC1R: front and center in the bright side of dark eumelanin and DNA repair. Int. J. Mol. Sci. 19, 2667. 10.3390/ijms19092667 30205559 PMC6163888

[B164] Takebayashi K. Chida K. Tsukamoto I. Morii E. Munakata H. Arnheiter H. (1996). The recessive phenotype displayed by a dominant negative microphthalmia-associated transcription factor mutant is a result of impaired nucleation potential. Mol. Cell Biol. 16, 1203–1211. 10.1128/mcb.16.3.1203 8622664 PMC231102

[B165] Tarafder A. K. Bolasco G. Correia M. S. Pereira F. J. C. Iannone L. Hume A. N. (2014). Rab11b mediates melanin transfer between donor melanocytes and acceptor keratinocytes via coupled exo/endocytosis. J. Invest. Dermatol. 134, 1056–1066. 10.1038/jid.2013.432 24141907

[B166] Tashiro A. Yuste R. (2004). Regulation of dendritic spine motility and stability by Rac1 and Rho kinase: evidence for two forms of spine motility. Mol. Cell Neurosci. 26, 429–440. 10.1016/j.mcn.2004.04.001 15234347

[B167] Thankachan J. M. Setty S. R. G. (2022). KIF13A-A key regulator of recycling endosome dynamics. Front. Cell Dev. Biol. 10, 877532. 10.3389/fcell.2022.877532 35547822 PMC9081326

[B168] Thomas A. J. Erickson C. A. (2008). The making of a melanocyte: the specification of melanoblasts from the neural crest. Pigment. Cell Melanoma Res. 21, 598–610. 10.1111/j.1755-148X.2008.00506.x 19067969

[B169] Tolmachova T. Ramalho J. S. Anant J. S. Schultz R. A. Huxley C. M. Seabra M. C. (1999). Cloning, mapping and characterization of the human RAB27A gene. Gene 239, 109–116. 10.1016/s0378-1119(99)00371-6 10571040

[B170] Tsukamoto K. Jackson I. J. Urabe K. Montague P. M. Hearing V. J. (1992). A second tyrosinase-related protein, TRP-2, is a melanogenic enzyme termed DOPAchrome tautomerase. Embo J. 11, 519–526. 10.1002/j.1460-2075.1992.tb05082.x 1537333 PMC556482

[B171] Van Gele M. Dynoodt P. Lambert J. (2009). Griscelli syndrome: a model system to study vesicular trafficking. Pigment. Cell Melanoma Res. 22, 268–282. 10.1111/j.1755-148X.2009.00558.x 19243575

[B172] Vega-Lopez G. A. Cerrizuela S. Tribulo C. Aybar M. J. (2018). Neurocristopathies: new insights 150 years after the neural crest discovery. Dev. Biol. 444 (Suppl. 1), S110–S143. 10.1016/j.ydbio.2018.05.013 29802835

[B173] Villareal M. O. Han J. Yamada P. Shigemori H. Isoda H. (2010). Hirseins inhibit melanogenesis by regulating the gene expressions of Mitf and melanogenesis enzymes. Exp. Dermatol 19, 450–457. 10.1111/j.1600-0625.2009.00964.x 19765058

[B174] Wakamatsu K. Ito S. (2023). Recent advances in characterization of melanin pigments in biological samples. Int. J. Mol. Sci. 24, 8305. 10.3390/ijms24098305 37176019 PMC10179066

[B175] Wang C. Liu Z. Huang X. (2012). Rab32 is important for autophagy and lipid storage in Drosophila. PLoS One 7, e32086. 10.1371/journal.pone.0032086 22348149 PMC3279429

[B176] Wasmeier C. Romao M. Plowright L. Bennett D. C. Raposo G. Seabra M. C. (2006). Rab38 and Rab32 control post-Golgi trafficking of melanogenic enzymes. J. Cell Biol. 175, 271–281. 10.1083/jcb.200606050 17043139 PMC2064568

[B177] Watanabe A. Takeda K. Ploplis B. Tachibana M. (1998). Epistatic relationship between Waardenburg syndrome genes MITF and PAX3. Nat. Genet. 18, 283–286. 10.1038/ng0398-283 9500554

[B178] Wei A. Yuan Y. Qi Z. Liu T. Bai D. Zhang Y. (2019). Instability of BLOC-2 and BLOC-3 in Chinese patients with Hermansky-Pudlak syndrome. Pigment. Cell Melanoma Res. 32, 373–380. 10.1111/pcmr.12748 30387913

[B179] Wei A. H. He X. Li W. (2013). Hypopigmentation in hermansky-pudlak syndrome. J. Dermatol 40, 325–329. 10.1111/1346-8138.12025 23668540

[B180] Williams A. B. Schumacher B. (2016). p53 in the DNA-Damage-Repair Process. Cold Spring Harb. Perspect. Med. 6, a026070. 10.1101/cshperspect.a026070 27048304 PMC4852800

[B181] Wilson D. S. Guenther B. Desplan C. Kuriyan J. (1995). High resolution crystal structure of a paired (Pax) class cooperative homeodomain dimer on DNA. Cell 82, 709–719. 10.1016/0092-8674(95)90468-9 7671301

[B182] Wilson S. M. Yip R. Swing D. A. O'Sullivan T. N. Zhang Y. Novak E. K. (2000). A mutation in Rab27a causes the vesicle transport defects observed in ashen mice. Proc. Natl. Acad. Sci. U. S. A. 97, 7933–7938. 10.1073/pnas.140212797 10859366 PMC16648

[B183] Witkop C. J. (1979). Albinism: hematologic-storage disease, susceptibility to skin cancer, and optic neuronal defects shared in all types of oculocutaneous and ocular albinism. Ala J. Med. Sci. 16, 327–330. 546241

[B184] Wolff K. (1973). Melanocyte-keratinocyte interactions *in vivo*: the fate of melanosomes. Yale J. Biol. Med. 46, 384–396. 4779127 PMC2592025

[B185] Wolff S. P. Jiang Z. Y. Hunt J. V. (1991). Protein glycation and oxidative stress in diabetes mellitus and ageing. Free Radic. Biol. Med. 10, 339–352. 10.1016/0891-5849(91)90040-a 1855674

[B186] Wu X. S. Rao K. Zhang H. Wang F. Sellers J. R. Matesic L. E. (2002). Identification of an organelle receptor for myosin-Va. Nat. Cell Biol. 4, 271–278. 10.1038/ncb760 11887186

[B187] Yamagata K. Nammo T. Sato Y. Saisho K. Shoda H. Fukui K. (2007). The HNF-1alpha-SNARE connection. Diabetes Obes. Metab. 9 (Suppl. 2), 40–45. 10.1111/j.1463-1326.2007.00773.x 17919177

[B188] Yamamoto O. Bhawan J. (1994). Three modes of melanosome transfers in Caucasian facial skin: hypothesis based on an ultrastructural study. Pigment. Cell Res. 7, 158–169. 10.1111/j.1600-0749.1994.tb00044.x 7971749

[B189] Yasumoto K. Takeda K. Saito H. Watanabe K. i. Takahashi K. Shibahara S. (2002). Microphthalmia-associated transcription factor interacts with LEF-1, a mediator of Wnt signaling. Embo J. 21, 2703–2714. 10.1093/emboj/21.11.2703 12032083 PMC126018

[B190] Young A. Wang Y. Ahmedli N. B. Jiang M. Farber D. B. (2013). A constitutively active Gαi3 protein corrects the abnormal retinal pigment epithelium phenotype of Oa1-/- mice. PLoS One 8, e76240. 10.1371/journal.pone.0076240 24098784 PMC3787026

[B191] Yu R. J. Van Scott E. J. (2002). Hydroxycarboxylic acids, N-acetylamino sugars, and N-acetylamino acids. Skinmed 1, 117–122. 10.1111/j.1540-9740.2002.01646a.x 14673337

[B192] Zhu Y. Li S. Jaume A. Jani R. A. Delevoye C. Raposo G. (2022). Type II phosphatidylinositol 4-kinases function sequentially in cargo delivery from early endosomes to melanosomes. J. Cell Biol. 221, e202110114. 10.1083/jcb.202110114 36169639 PMC9524207

[B193] Zucca F. A. Capucciati A. Bellei C. Sarna M. Sarna T. Monzani E. (2023). Neuromelanins in brain aging and Parkinson's disease: synthesis, structure, neuroinflammatory, and neurodegenerative role. IUBMB Life 75 (1), 55–65. 10.1002/iub.2654 35689524 PMC10084223

